# From Microtiter Plates to Droplets—There and Back Again †

**DOI:** 10.3390/mi13071022

**Published:** 2022-06-28

**Authors:** Thomas Henkel, Günter Mayer, Jörg Hampl, Jialan Cao, Linda Ehrhardt, Andreas Schober, Gregor Alexander Groß

**Affiliations:** 1 Leibniz Institute of Photonic Technology, Leibniz-IPHT, Albert-Einstein-Str. 9, 07745 Jena, Germany; thomas.henkel@leibniz-ipht.de (T.H.); guenter.mayer@leibniz-ipht.de (G.M.); 2 Department of Nano-Biosystem Technology, Institute of Chemistry and Biotechnology, Technical University Ilmenau, Prof.-Schmidt-Str. 26, 98693 Ilmenau, Germany; joerg.hampl@tu-ilmenau.de (J.H.); andreas.schober@tu-ilmenau.de (A.S.); 3 Department of Physical Chemistry and Microreaction Technologies, Institute of Chemistry and Biotechnology, Technical University Ilmenau, Prof.-Schmidt-Str. 26, 98693 Ilmenau, Germany; jialan.cao@tu-ilmenau.de (J.C.); linda.ehrhardt@tu-ilmenau.de (L.E.)

**Keywords:** microfluidics, mems device, parallel to serial, droplet aspiration, robotic, transfer, re-formatting, transfer operation, liquid handling, screening, microtiter-plate, MTP, fluid connector

## Abstract

Droplet-based microfluidic screening techniques can benefit from interfacing established microtiter plate-based screening and sample management workflows. Interfacing tools are required both for loading preconfigured microtiter-plate (MTP)-based sample collections into droplets and for dispensing the used droplets samples back into MTPs for subsequent storage or further processing. Here, we present a collection of Digital Microfluidic Pipetting Tips (DMPTs) with integrated facilities for droplet generation and manipulation together with a robotic system for its operation. This combination serves as a bidirectional sampling interface for sample transfer from wells into droplets (w2d) and vice versa droplets into wells (d2w). The DMPT were designed to fit into 96-deep-well MTPs and prepared from glass by means of microsystems technology. The aspirated samples are converted into the channel-confined droplets’ sequences separated by an immiscible carrier medium. To comply with the demands of dose-response assays, up to three additional assay compound solutions can be added to the sample droplets. To enable different procedural assay protocols, four different DMPT variants were made. In this way, droplet series with gradually changing composition can be generated for, e.g., 2D screening purposes. The developed DMPT and their common fluidic connector are described here. To handle the opposite transfer d2w, a robotic transfer system was set up and is described briefly.

## 1. Introduction

In the recent decade, droplet-based microfluidics has attracted a reasonable interest, especially in the area of life science research [1,2,3,4,5,6,7]. One key advantage is the scientifically relevant information which can be gained by consuming only a very low volume of samples. On the other hand, the droplet-based techniques enable the processing of huge numbers, which qualifies this technique for high throughput experimentation [8,9,10,11,12]. Channel-confined droplet microfluidics takes advantage of the immiscibility of carrier and sample phases [13]. The sample droplets are separated in a serial manner in a confining channel or tube [14,15]. In this way, hundreds to thousands of droplets can be generated, handled, and analyzed efficiently. The so-called “segmented flow” technique has been successfully introduced for a wide range of applications, such as: (a) concentration-dependent synthesis of different types of nanoparticles [16]; (b) cultivation and susceptibility studies of microorganisms [17,18], in particular, for measuring highly-resolved dose/response functions in micro-toxicology screening [19,20]; (c) for the screening of two- or three-dimensional concentration spaces and of stochastic confinement strategies to explore rare microorganisms [21,22]; (d) the synthesis of polymeric micro-rods [23], to name some exemplary applications. 

However, even if there are many significant advantages in terms of the efficient realization of screening experiments in the sub-µL volume range, the channel-confined droplet techniques are mostly found in the academic development phase. The established standard in the life science industry still consists of robots and tools optimized for Microtiter-plate (MTP) use, e.g., pipetting and handling robots, plate washers and dispensers, readers, incubators, and some more. Hence, the interface between MTP-based processing and droplet-based processing seems to be an essential requirement to combine the advantages of both worlds. Nevertheless, some solutions targeting the problem of robotic sample to droplet transfer and vice versa have been published before [24,25,26,27,28,29]. We described earlier the basic operation of plate to droplet transfer using a looped tubing [30]. Here, a PTFE-tube-based system was introduced, capable to aspirate droplet sequences from a sample source after immersion. For the overall transfer process, different supporting peripherals such as a valve operation, x-y-z positioning system, and photometric sensors are required, and were described earlier as well [30]. Here, we present a novel glass-based microsystems engineering solution for the aspiration of sample fluids, e.g., from a 96-MTP well. The Digital Microfluidic Pipette Tips (DMPT) are available in some variants to allow the different assay protocols. To solve the challenge the other way around, a commercial robot system was adapted to transfer single droplets from tube-based sequences into individual MTP wells. 

## 2. The Digital Microfluidic Pipette Tips (DMPTs)

### 2.1. Device Layout

**Figure 1:**
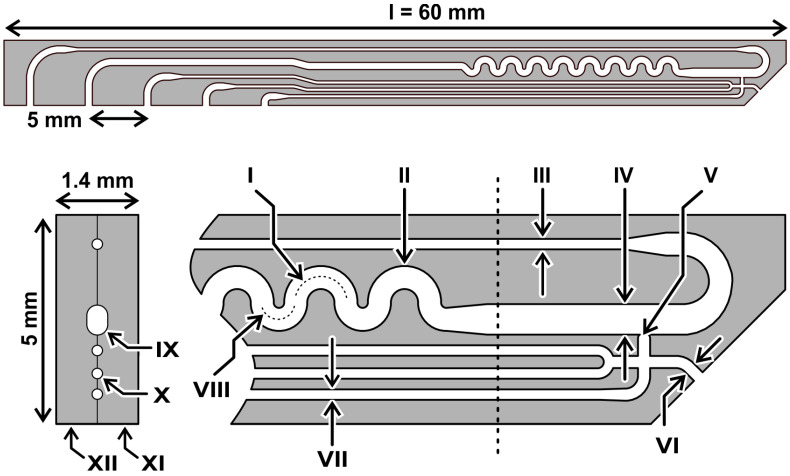
Dimension and layout of a DMPT. I: r = 600, d = 200; II: w = 550, d = 200; III: w, d = 100; IV: w = 730, d = 200; V: nozzle diameter appr. el 220, high 100; VI: w = 290, d = 100 VII: w, d = 100; VIII: r = 400; IX: d = 200; X: d = 100; XI = 1st half shell; XII = 2nd half shell (unit = µm, r = radius, w = width, d = etching depth).

**Figure 2:**
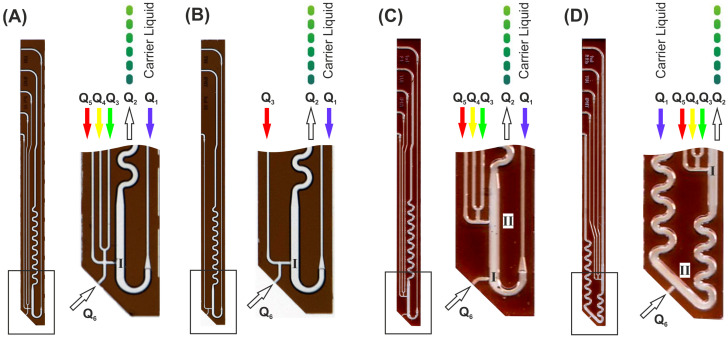
Images of different transfer devices designed for changed droplet formation and effector addition order. Droplets are generated at position I. The addition of the effector or dilution fluids to preformed droplets takes place at position II. Boxed areas are shown as magnification. (**A**) The aspirated sample (Q_6_) can be mixed with three different effector/dilution liquids (Q_3–5_) before the stream is injected into the carrier medium for droplet generation. (**B**) This device was designed for single dilution (Q_3_) of the aspirated sample (Q_6_) before the droplet generation. (**C**) This device allows the formation of plain sample droplets (Q_6_) before premixed effector or dilution liquids (Q_3–5_) are added. (**D**) The device was designed for the initial generation of premixed effector/dilution liquid droplets (Q_1–3_) prior to the addition of the aspirated sample (Q_6_). Flow rate $Q=\frac{dV}{dt}\;\left[\frac{µL}{min}\right]$.

The dimensions of the DMPTs were designed to fit in size into a deep-well 96-well microtiter plate (see Figure 1). Therefore, two micro structured, mirror-symmetrical glass pieces bearing the channel half shells were produced by use of a lithographic photo mask and subsequent isotropic wet etching technique on a glass wafer basis. The microstructured wafers were bonded together to a monolithic device using a poly-silicon bonding technique. After dicing and finishing, the devices, as shown in Figure 2, were received. The device dimension of 60 mm length 5 mm width was chosen with respect to the MTP deep well size. The thickness of the bonded device was given by the used glass wafer thickness and led to an overall thickness of 1.4 mm. The device was fabricated by two subsequent etching steps, leading to 100 µm and 200 µm etching depth in the half shells. After half-shell bonding, the received channel height results were twice the size of these values. In Figure 1, the device dimensions are given in detail. The nozzle (V) and channel dimension (IV) were designed to produce droplets of about 500 nL volume in the main channel. Interface to the media feeding- and droplet-storage-tubings are the upper side channels of the DMPT, with the dimensions as shown in the cross section of Figure 1. All DMPT-layouts were designed to produce finally stable droplets in subsequent storage tubings as rod-shape droplets with about 4 mm length.

### 2.2. Device Function and Variants for Different Assay Requirements 

The operation principle of the device makes use of the volumetric balance principle. If the volume flow rate $Q=\frac{dV}{dt}\;\left[\frac{µL}{min}\right]$ which leaves the device (Q_2_) is greater than the volume flow rates which enter the device (Q_1_, Q_3–5_), the difference will be aspirated in a passive way through the immersed channel opening (Q_6_). Figure 2 shows the corresponding channels and their flow rate identifiers.

Screening assays usually are designed as dose/response-type experiments [14]. Therefore, sample series with varying composition are required. Thus, droplet-based systems need to generate sequences with gradually changing droplet compositions regarding the concentration of an effector under investigation. From an operational point of view, the droplets must be diluted by different media during formation [31,32,33]. The final droplet should have a constant volume, whereas all other assay components such as substrate concentration or cell density must be equal in all droplets. Depending on the desired assay protocol, different assay components must be added during the droplet formation. To solve this problem, we built different DMPT layouts varying in number and order of complementary feed channels. Figure 2 shows the four obtained variants with different channel arrangement in the tip. The device in Figure 2A allows the aspirated sample to be mixed with up to three different fluids (Q_3_–Q_5_) before the fluids enter the main channel through the nozzle at position (I). In the main channel, the streaming carrier medium (Q_1_) leads to the desired droplet formation. Version (B) has only one supporting channel (Q_3_) which allows, e.g., a gradually diluted aspiration of the sample fluid before droplet generation. In version (C) and (D), the order of the initial sample droplet formation and supporting assay media addition is varied. Using layout (C), the aspirated sample liquid is converted into droplets at position I. The addition of further assay components take place at a second nozzle II in the subsequent channel path immediately before the supplemented droplets enter the mixing meander. Version (D) allows the opposite assay operation: initial droplet formation of assay components before the aspirated sample is added. Here, a second mixing meander was placed between the initial droplet formation nozzle (I) and the supplementing nozzle (II). However, different assay modes can be realized by selecting the appropriate DMPT variant. The devices can even be used if not all channels are required. In this case, unused channels should be filled with fluorinated carrier medium and sealed at the end. 

### 2.3. Surface Functionalization and Operation

Precondition for channel-confined microfluidics are the appropriate surface properties of the used channels. The wettability between the sample phase and the channel surface must be minimized, while the wettability of the carrier phase must be maximized for stable droplet processing. In case of aqueous sample droplets and fluorinated carrier media, the channel surface needs to be hydrophobic or, at best, fluorophilic. For HO-rich glass surfaces, the chemical surface modification by perfluorinated octadecyl-trichloro-silanes (F-ODT) allows the conversion of the hydrophilic surface into a fluorophilic surface [34,35,36]. We kept the primed and dried devices overnight in a solution of 5% (*v*/*v*) F-ODT in a mixture of THF and 10% (*v*/*v*) anhydrous Ethanol at 40 °C. Following, the device was washed with anhydrous Iso-Propanol and kept at 80 °C in Iso-Propanol in a closed vial for another 2 h. Finally, the devices were dried by blowing with compressed air before tempering at 180 °C in vacuum for 2 h. In this way, fluorophilic properties at the inner and outer surfaces were received. At the outer surface, contact angles of about Θ ≥ 160° were obtained, whereas the measurement of the contact angle for fluorinated oil (PP9) could be estimated to be less than Θ ≤ 5° because of the excellent wettability and widespread sessile droplets. However, the stability of the fluorophilic surface properties is limited and may become lost if the devises are used with alkaline solutions or stored in aqueous media for extended periods. The stabile hydrophobic outer device surface is important for a proper sample dewetting after pulling up the DMPT from the immersed sample or washing fluid. 

**Figure 3:**
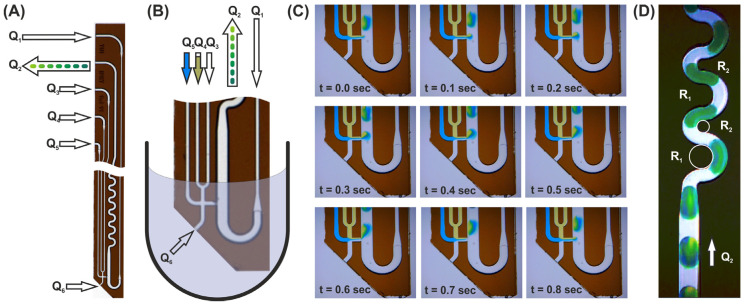
(**A**) Image of the glass made transfer tool. The arrows represent the different flow rates (Q_i_) for operation. The device is 16 mm long, 6 mm wide, and possess a thickness of about 1.4 mm. (**B**) Immersed tool tip for sample aspiration (schematic). (**C**) Image series of the droplet generation process. The mass balance of the actively controlled flow rates Q_1–5_ determine the sample aspiration flow rate V_6_. Sample volume will be aspirated as long as the sum of the derived flow rates Q_1_ + Q_3_ + Q_4_ + Q_5_ are less than the aspiration flow rates Q_2_. The sample flow rate can then be calculated by Q_6_ = Q_2_ − (Q_1_ + Q_3_ + Q_4_ + Q_5_). V_1_ is the flow rate which provides the immiscible separation medium. The ratio between Q_1_/(Q_3_ + Q_4_ + Q_5_ +Q_6_) determines the droplet distances and is usually set to about 1. (**D**) Image of mixing droplets as they pass through the meandered channel. The meander is designed with two different radii (R_1_ = 2 × R_2_) in the channel curvatures for fast dean mixing. (Further details see text).

In Figure 3, the droplet aspiration of a F-ODT modified DMPT device is shown; Figure 3A,B depicts the applied fluid streams after immersion into the sample fluid (B). The image series (C) shows the droplet formation processes during aspiration of an aqueous sample and simultaneous addition of two exemplary colored effector solutions (Q_5_ = blue and Q_4_ = yellow). The following flow rates were applied: Q_1_ = 50 µL/min, Q_2_ = −100 µL/min, Q_3_ = 0 µL/min, Q_4_ = 12.5 µL/min, and Q_5_ = 12.5 µL/min. In this case, about 500 nL droplets composed of 50% aspirated sample, 25% blue (Q_5_), and 25% yellow effector solution (Q_3_) were received. Corresponding videos (Video_S1 and Video_S2) can be found in the SI. Changing the flow rates Q_5_ and Q_3_ over time leads to the generation of the above-mentioned droplet sequences with gradually changing effector concentration. The operation of variant (B), as shown in Figure 3C, is also shown in the SI (Video_S3). Image (D) shows the mixing behavior of the generated droplets. Along the straight channel, the inhomogeneously formed droplet is not mixed convectively because of the symmetric flow field [13]. To overcome this problem, a meandering channel with two different radii was used (Figure 3D). In this way, the mixing time is significantly reduced by induced Dean-flow vortices. Even at flow rates of about 50 µL/min, the optical analyses show a homogenous coloring of the droplets after passing 4–5 meanders. A concerning video can be found in SI (Video_S4). At higher flow rates, faster mixing can be achieved [37]. 

## 3. DMPT-Fluid Connector and Robotic Workflow

From a holistic perspective, the interface between microfluidic systems and supporting tubing is essential, though it is not mentioned in most publications. However, the robust operation of microfluidic devices is highly dependent on the peripherals that are functionally required. In particular, the interface between chip and tubings is a critical element which is responsible for failure in many cases. Besides the fluidic features, the main issue for a connector is easy and safe handling during assembly and disassembly. 

### 3.1. Fluid Connector for the DMPTs and Other Fluidic Devices

**Figure 4:**
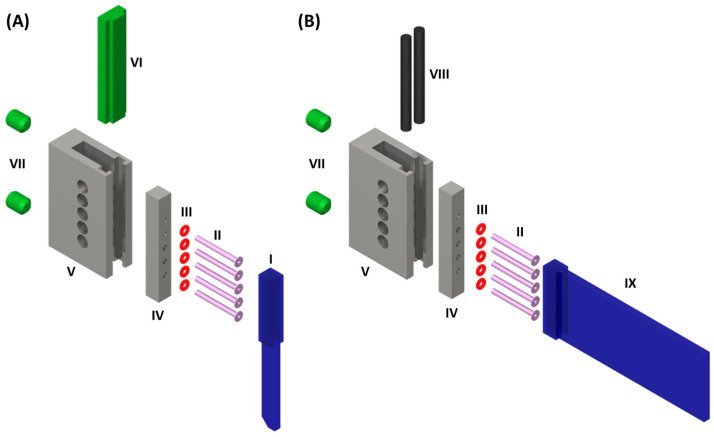
Exploded view scheme of the connector interfacing fluidic devices and the tubing: (**A**) Fluid connector assembly for DMPT. (**B**) Fluid connector assembly for other planar microfluidic devices. (I) Droplet sampling tool; (II) Flanged PTFE-Tubing (1/16″ OD, 0.5 mm ID, ca. 4 mm flange radius); (III) Rubber O-rings; (IV) Aluminum crossbars with through holes for the tubing; (V) Aluminum main frame with side openings for visual adjustment of tube to device alignment. Tapped holed for the grub screws (VII) and groove inside for the plastic support (VI) for the fluidic device. (VIII) Rubber sealing cord, (IX) planar microfluidic chip with fluid inlets on the face-side. The fluidic devices (I) and (IX) are planar due to their manufacturing process and must be supplemented by rectangular glass plates to fit in the connector. The supporting glass plates were glued onto the outsides of the fluidic devices in the area using a photo adhesive.

**Figure 5:**
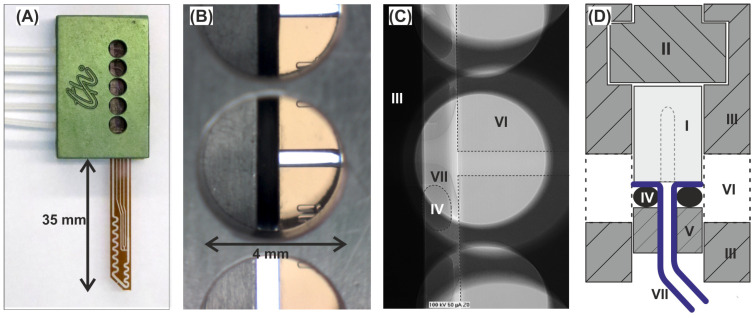
Connector for the DMPTs: (**A**) Image of a DMPT assembled within the connecting frame equipped with five tubes (Q_1–5_). The aluminum main frame contains five inspection openings which allow the alignment of the DMPT relative to the flanged tubing. (**B**) Magnification of an inspection opening showing the glass device with channel and O-ring gasket bearing the flanged tubing. (**C**) X-ray image of the in (**B**) shown assembly; (**D**) Cross-sectional scheme perpendicular to the top view showing following elements: I: DMPT; II: polymeric support for the fluidic device; III: main frame with inspection openings; IV: O-rings; V: crossbar with through holes for the tubing; VI: inspection openings for aligning adjustment; VII: flanged tubing.

We have designed a general connector to connect the microstructured glass devices such as the DMPTs with the required tube connections. Figure 4A shows the function of the developed connector. The core function of the connector is to provide a tight and secure connection between the tubing and the brittle glass device. It is crucial to ensure that the droplets generated inside the device are not disturbed during the transition into the tubing. For this purpose, the flanged ends of the tubing (II) are pressed onto the upper channel opening of the DMPT (I) supported by elastic O-rings (III), which are mounted on the tubing and placed behind the flange. The O-ring-supported tubings are plugged through a crossbar (IV), which will be forced by screws (VII) against a polymeric support (VI), which will be inserted in the main frame (V) before. In this way, the brittle glass DMPT comes only into contact with the elastic and polymorphic parts and is protected against mechanic forces. Figure 4B illustrates the connector assembly for rectangular fluidic device. In this case, elastic rubber sealing cords (VIII) are placed in the groves of the main frame (V) between the device (IX). In this way, the mounted device is only in contact to the rubber sealing cords and the O-ring-supported tubing. For the axial alignment of the devices channels in (I) and (IX) relative to the tubing (II), the main frames (V) are equipped with five inspection ports for optical alignment. In Figure 5A the assembled connector is depicted. (B) shows the view through an aligning hole, (C) the X-ray image of the connection between the flanged tubing and the device. (D) shows the cross-sectional scheme of the assembled connector.

### 3.2. Periphery and Workflow for the Digital Microfluidic Pipetting Tips

**Figure 6:**
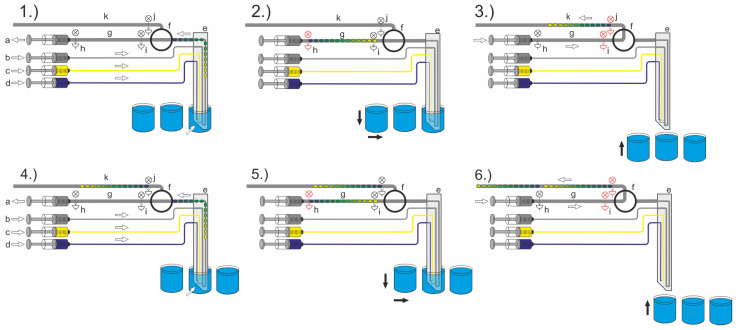
Scheme of the overall droplet sequence aspiration system workflow. (The process step order (1.–6.) is discussed in the text).

The entire droplet aspiration process to generate the desired droplet sequences from a sample source requires the coordination of different fluidic and mechanical operations. The general setup, including the required peripheral tools, have been previously described. However, the setup scheme and process flow are shown in Figure 6. At least one four-channel syringe pump (a–d), a droplet-compatible valve (f), and the DMPT (e) must be assembled and controlled. The droplet-compatible valve (f) has a key function for the aspiration process. The depicted process flow shows an assay which makes use of two effector solutions which will be added during sample aspiration (c, d). Hence, the syringe pump is loaded as follows: (a) aspiration syringes filled to about 5% volume with carrier medium, (b) carrier medium dosage syringe, (c) effector solution one, (d) effector solution two. After initialization of the system, namely loading and priming of all channels, the DMPT is moved into the first sample (l) by the x-y-z robot. Here, the droplet sequence aspiration process takes place. Depending on the desired assay protocol, the droplet sequence may be aspirated with different amounts of the effectors (syringe c, d). If the sequence aspiration is completed, the flow rates of the carrier medium delivering syringe (b) and aspiration syringe (a) are set to equal flow rates (Figure 3 Q_1_ = Q_2_) with an opposite direction. In this way, no more droplets are generated inside the device tip, but the carrier phase passes the nozzle and moves the aspirated sequence along the way through the valve (Figure 6, (1.)). The intermediate storage tubing length (g) determines the length of the aspirated droplet sequences and must be considered for the aspiration procedure. To handle the intermediate storage, two photoelectric sensors (h, i) are placed on the tubing. The droplet sequence aspiration is triggered with the help of sensor (h). If the sensor (h) detects the first droplet, the syringe pumps (a, b) are stopped, and the valve (f) will be switched to the second position (Figure 6, (2.)). Then, the sequence is fed through the valve in the direction of the storage and incubation tube (k) using the syringe (a). The photoelectric sensors (i and j) are used to monitor the transport through the valve (Figure 6, (3.)). If sensor (j) detects the last droplet, the syringe is stopped, and the valve (f) is switched back to the initial aspiration position. Simultaneously, the DMPT is moved forward to the next MTP-well position (m) for the next sample aspiration, and the aspiration flow rate regime is restarted (Figure 6, (4.)). The aspiration process starts from the beginning (Figure 6, (5.) + (6.)). We made use of an additional washing step before immersion into the next well. Therefore, the device was immersed into a well filled with washing liquid to prevent crosstalk between the samples. 

## 4. Characterization of the DMPT 

**Figure 7:**
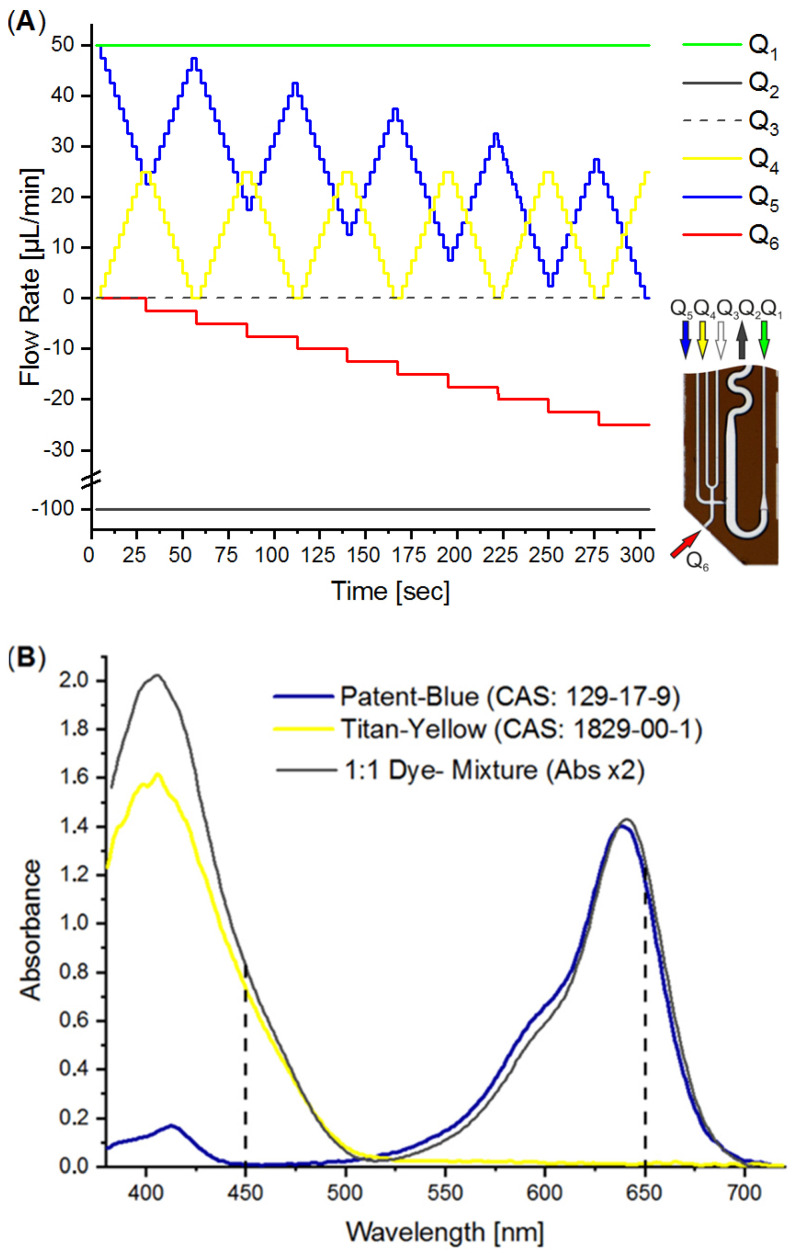
(**A**) Flow rate program for the generation of droplet sequence covering a 2D concentration space. (**B**) UV-Vis spectra of the used indicator dye solutions and mixture. Flow rate $Q=\frac{dV}{dt}\;\left[\frac{µL}{min}\right]$.

**Figure 8:**
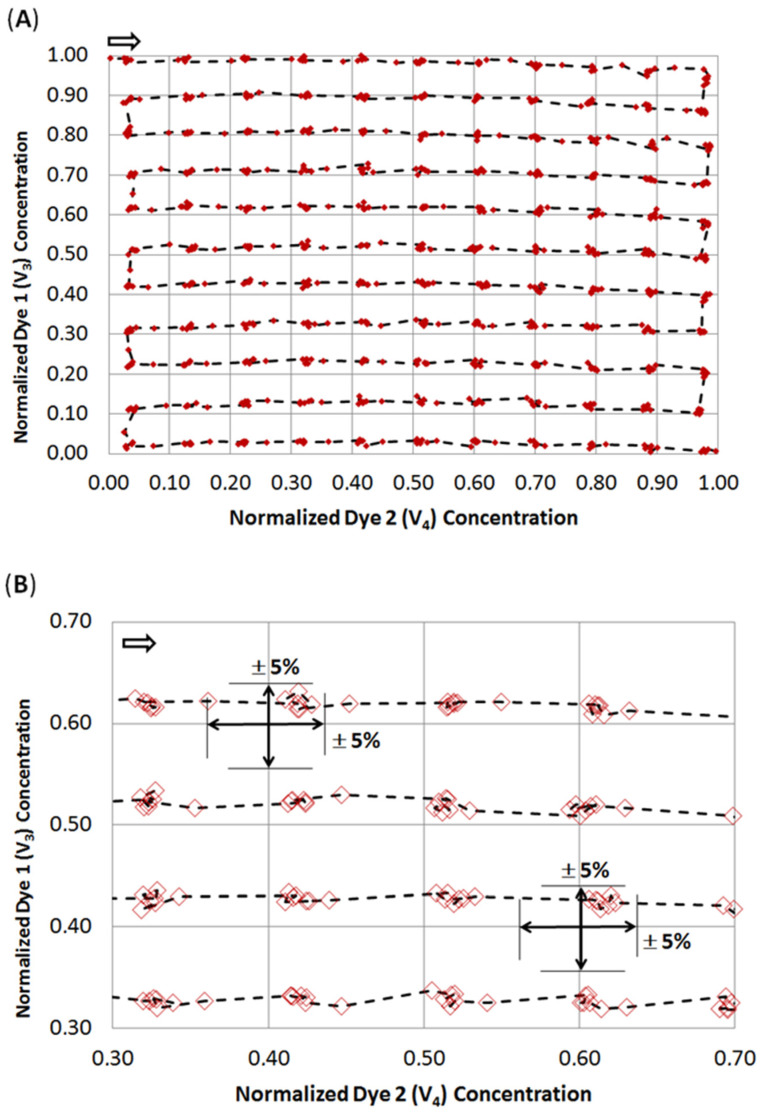
Measured droplet composition screening a 2D concentration space. The droplets were composed of two different dye solutions and diluted by the aspirated sample. The stepwise concentration change set by the flow rate program (Figure 7A) can be clearly seen in the diagram (**A**). The arrow in (**A**) indicates the temporal order of generated droplets regarding the flow rate program (Figure 7A). The deviation or the received droplet composition from the theoretical one is less than 5% for 97% of the droplets. (**B**) Excerpt of the diagram (**A**) showing the measured droplet composition in greater detail.

The DMPT device (Figure 2A) was tested by aspirating water from an MTP source with the parallel addition of two differently colored effector solutions as an example. We made use of an earlier-described colorimetric characterization method to determine the accuracy of the received droplet composition [14,38]. For this purpose, titanium yellow and patent blue dye solutions were prepared in a 50% water/DMSO mixture with an absorbance of about 1.5. The DMSO solution was chosen to mimic stock solutions for screening compounds. The colored solutions were filled into the syringes and used for the flow rates Q_3_ and Q_4_ (see Figure 7A). After priming the fluidic system, the DMPT was immersed into the MTP-well filled with DI-water and the syringe–pump-program (Figure 7A) was initiated. The syringe–pump-program was developed to cover a 2D concentration space of the both dyes and successive dilution by the aspirated water. To achieve a concentration resolution of 10%, 11 different concentration steps (0%, 10%…100% of max. dye concentration) were programmed for Q_4_ and Q_5_, resulting in 121 combinations. This program was chosen to prove the accuracy of the droplet formation of the DMPT, as Figure 2A illustrates. Other screening protocols using different operation modes and DMPT variants should show comparable results in terms of fluidic precision. The syringe pump program is shown in Figure 7A. The difference between the aspiration flow rate Q_2_ (−100 µL/min) and the carrier medium flow rate Q_1_ (50 µL/min) determines the volume flow rate which must be aspirated from the sample (Q_6_) or substituted by the feeding flow rates Q_3_ and Q_4_. Nevertheless, the received droplet sequence possesses equal volume fractions of spacer- (carrier) and sample-droplets. Under these conditions, droplets are formed with a frequency of about 0.4 Hz. To verify the droplet composition accuracy determined by the DMPT and supporting syringe pumps, the received droplet content was analyzed by inline UV-Vis spectroscopy. In Figure 7B, the droplet born UV-Vis spectra of the used dye solutions are shown. The appropriate volumetric fractions of the different solutions in the droplets were calculated using the absorbance at 450 and 650 nm and normalized to 0–1.0. The received data were plotted in a 2D scatter plot and connected by a dashed line according to their order. In Figure 8, the received 2D concentration space is shown. The grid intersections represent the theoretical concentration set by the applied flow rate program. The red points show the measured compositions. The dashed line indicates the order of droplet formation starting at the arrow. At least 852 droplets were generated within 300 sec. The analysis of the deviation between the theoretical and received droplet composition shows that the more than 97% of the received droplets deviate less than ±5% from the expected values (see Figure 8B). The least 3% of droplets are the intermediate droplets which are formed between two composition points. However, the step-wise concentration change set by the flow rate program is clearly visible in Figure 8A and proves the suitability of the DMPT for screening applications. The comparison between the different devices of one layout as well as between the device of the variants in Figure 2C,D revealed comparable results for the 2D concentration space characterization. However, the used flow rate programs must be adjusted carefully to synchronize the droplet generation and merging in these cases (data not shown). 

## 5. Droplet to Vial Transfer 

**Figure 9:**
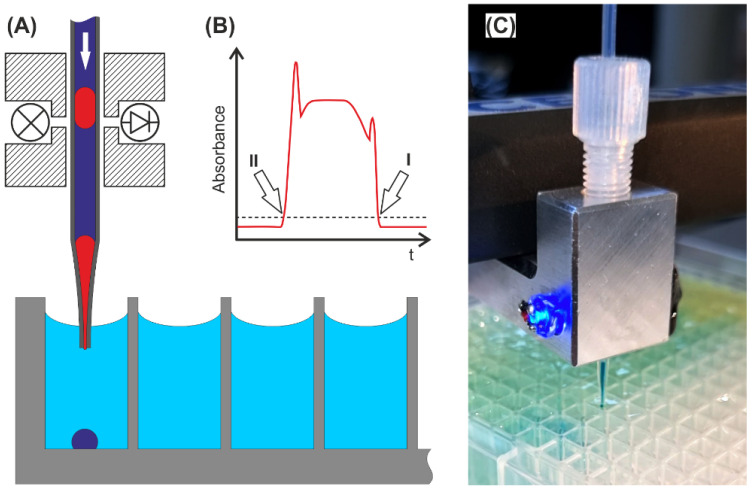
Droplet to vial transfer: (**A**) Schematic droplet dispensing into MTP wells. The tube and photoelectric sensor were mounted in a frame. The tubing was stretched to form a tip. (**B**) Signal of the photoelectric sensor (LED 470 nm, Photodiode) during droplet passage. If the absorbance signal exceeds the set threshold, the droplet front (I) and end (II) can be detected and used as trigger signal for the robotic movement. (**C**) Image of the transfer tool dispensing droplets into a prefilled 384-well plate. (See Video_S6).

Droplet sequences used for sub-µL screening purposes may need to be transferred back into cavities of larger volumes for further processing. Therefore, the channel- or tube-confined sequentially arranged droplets must be dispensed individually into arrayed vials of, e.g., MTPs. To solve this task, the tube-based droplet sequence must be pumped to the tube-end (tip), and the exiting droplets must be collected individually inside a target well. Some problems must therefore be solved by engineering means: control of the wetting behavior of the spilling droplet between tip and target well, handling the carrier medium and the coordination of the flow rate-dependent droplet frequency and robotic tip move. We combined a commercially available robotic system with a syringe pump and a photoelectric sensor for droplet detection. The syringe pump was loaded with the carrier medium and connected to one end of the droplet bearing tube coil. The other end of the tube was equipped with the photoelectric sensor and mounted to the positioning robot (see Figure 9). In this way, the position of the robotic tip can be triggered by the photoelectric sensor signal, and each spilling droplet can be dispensed into an individual MTP-well. The precise transfer of low volumes requires contact dispensing. To circumvent the wettability problem during the liquid transfer of the exiting droplets and carrier medium from the tube tip into the target well, the target well was prefilled with aqueous buffer medium. Without prefilled target wells, there is a risk of failing dispensing, because the exiting droplet adheres to the tubing tip and will be moved to the next well. Under this operation regime, the carrier medium between the droplets will be dispend into the wells as well. However, the used fluorinated oils are inert for the most biological applications. The carrier medium possesses a significantly higher density in comparison to aqueous solutions and will be found at the bottom of the well. If it is expected that the carrier phase will interfere with subsequent MTP-based screening steps, a partial volume transfer into a fresh MTP-plate will be required to separate the aqueous supernatant from the carrier phase. The droplet transfer rate is determined by the motion speed of the positioning robot, the distance of the droplets inside the tube, and the sensorics droplet detection. Once the first droplet is detected, the tip must be moved into the target well. After dispensing of the first droplets, the detection of the following droplet triggers the movement into the next well. To ensure accurate and error-free transfer, the flow rate must be adjusted to match the timed movement process. Hence, the tip movement from well to well must proceed in the same time interval. However, we used droplet sequences with a distance between droplets of about three times the droplet length. Using a 0.5 mm ID-Tubing, 500 µL droplets of about 8 mm length and a 100 µL/min syringe pump feeding rate leave the tube tip at about 2 Hz. In the SI, a video (Video_S5) is given, showing the transfer of a sequence of colored droplets into a prefilled (8 µL) 386-well plate. In this case, the transfer of 386 droplets into the plate requires about 12 min.

## 6. Conclusions

We developed Digital Microfluidic Pipette Tips (DMPTs) which allow the formation of droplet-based screening sequences in sub-µL volume scale, aspirated from MTP-based solutions. The device was made by glass micro system engineering. Four different DMPT variants were developed to enable different procedural assay protocols. Hence, the order of droplet generation and effector addition can be adjusted. The fluidic accuracy was proved by inline spectroscopy analysis of an exemplary two effector screening covering a 2D space in 10% steps. The desired droplet composition was achieved with a precision of +−5%. The reverse transfer of droplets into MTPs was realized by implementation of a customized robot. The system was proven by a 100% droplet transfer into a 384-well plate within 12 min. The presented technical solution offers the opportunity to combine microfluidic screening processes with traditional MTP-based screening processes in unified workflows. This enables one to combine the advantages of both worlds.

We gratefully acknowledge the support of the publication costs by the open access publication fund of the Technische Universität Ilmenau.

## Supplementary Materials

The following are available online at https://www.mdpi.com/article/10.3390/mi13071022/s1, Video_S1: DMTP Tip Type-A, Video_S2: DMTP-Tip and channels Type-A, Video_S3: DMTP-Tip and channels Type-B, Video_S4: Droplet mixing, Video_S5: DMTP Type-C, Video_S6: Droplet transfer into 384-plate.
